# Optimization and Analysis of Surface Roughness, Flank Wear and 5 Different Sensorial Data via Tool Condition Monitoring System in Turning of AISI 5140

**DOI:** 10.3390/s20164377

**Published:** 2020-08-05

**Authors:** Mustafa Kuntoğlu, Abdullah Aslan, Hacı Sağlam, Danil Yurievich Pimenov, Khaled Giasin, Tadeusz Mikolajczyk

**Affiliations:** 1 Technology Faculty, Mechanical Engineering Department, Selcuk University, Selçuklu, 42130 Konya, Turkey; hsaglam@selcuk.edu.tr; 2 Engineering and Architecture Faculty, Mechanical Engineering Department, Selcuk University, Akşehir, 42550 Konya, Turkey; aaslan@selcuk.edu.tr; 3 Department of Automated Mechanical Engineering, South Ural State University, Lenin Prosp. 76, 454080 Chelyabinsk, Russia; danil_u@rambler.ru; 4 School of Mechanical and Design Engineering, University of Portsmouth, Portsmouth PO1 3DJ, UK; Khaled.Giasin@port.ac.uk; 5 Department of Production Engineering, UTP University of Science and Technology, Al. prof. S. Kaliskiego 7, 85-796 Bydgoszcz, Poland; tami@utp.edu.pl

**Keywords:** Tool Condition Monitoring, flank wear, surface roughness, cutting force, vibration, acoustic emission, temperature, motor current

## Abstract

Optimization of tool life is required to tune the machining parameters and achieve the desired surface roughness of the machined components in a wide range of engineering applications. There are many machining input variables which can influence surface roughness and tool life during any machining process, such as cutting speed, feed rate and depth of cut. These parameters can be optimized to reduce surface roughness and increase tool life. The present study investigates the optimization of five different sensorial criteria, additional to tool wear (*V_B_*) and surface roughness (*Ra*), via the Tool Condition Monitoring System (TCMS) for the first time in the open literature. Based on the Taguchi L_9_ orthogonal design principle, the basic machining parameters cutting speed (*v_c_*), feed rate (f) and depth of cut (*a_p_*) were adopted for the turning of AISI 5140 steel. For this purpose, an optimization approach was used implementing five different sensors, namely dynamometer, vibration, AE (Acoustic Emission), temperature and motor current sensors, to a lathe. In this context, *V_B_*, *Ra* and sensorial data were evaluated to observe the effects of machining parameters. After that, an RSM (Response Surface Methodology)-based optimization approach was applied to the measured variables. Cutting force (97.8%) represented the most reliable sensor data, followed by the *AE* (95.7%), temperature (92.9%), vibration (81.3%) and current (74.6%) sensors, respectively. RSM provided the optimum cutting conditions (at *v_c_* = 150 m/min, *f* = 0.09 mm/rev, *a_p_* = 1 mm) to obtain the best results for *V_B_*, *Ra* and the sensorial data, with a high success rate (82.5%).

## 1. Introduction

Sensors are devices that measure a certain value and convert them into electrical signals. The main working principle of sensors is to convert physical changes into electrical quantities, and match mechanical systems with electrical systems. Thus, they ensure that a physical change in a system can be detected, measured and transferred to the environment, where it will be evaluated by the observer. As a result, they work as a source of information about a system. In machining, the aim is to obtain a stable and reliable production using various sensors. It is desired to prevent unexpected losses by monitoring the manufacturing process with high sensitivity sensors. Various breakdowns and accidents cause the halting of the production, leading to increased costs and lower efficiency [1]. Such accidents lead to idle time and increased waste material. The sustainability of a production chain becomes very important in mass production, especially in today’s competitive manufacturing industry [2]. For this reason, there is a need for a TCMS (Tool Condition Monitoring System) that works as a source of information related to machine tools and processes [3,4]. Therefore, the integration of various types of sensors into machine tools is essential for them to provide their analysis and optimal values under different conditions of product manufacturing.

Machining processes create various physical effects caused by high speeds. During the cutting process, chip formation occurs in the metal, which undergoes high plastic deformation. There is also noise and vibration in the tool and the machine, and temperature and pressure changes at the tool–workpiece chip interface. The sudden increase or decrease of the mentioned variables indicates that the steady state of the machining can change. It may not be sufficient to use a single sensor in the processing environment, where many parameters are constantly changing. Therefore, there is a need for reliable and sensitive sensors that will provide instant data about the process, and verify each other in predicting variables [5,6].

The changes that occur during the manufacture of a component are derived from the complex structure of the process and the dynamic processing conditions. Considering the inhomogeneity in the material structure and the lack of rigidity in the system, unexpected developments occur inevitably. The repetitive loads on the tool during metal cutting lead to the deprivation of material and the change of tool geometry over time [7,8]. The resulting deformation causes a gradual breakdown of the tool, and deteriorates its cutting [9]. The main goal is to give a warning before a certain level of tool wear is reached that could cause sudden tool failure [10]. During the machining, many parameters that change with the increase of wear, such as cutting forces, cutting temperatures, vibrations in the tool, the sound, chip and current, can be used as information sources to determine the severity of tool wear. Therefore, factors affecting the machining economy and productivity, such as tool life, chip control, idle time of the machine, waste material, power and time, can be controlled at the desired level, especially the final targets (production of the workpiece at desired tolerances and surface quality) [11,12]. The main problem that should be solved here is to observe the behavior of different cutting variables simultaneously in the complex nature of machining operations.

Balazinski et al. [13] used a system of sensors to monitor tool wear based on the cutting forces in turning, and various artificial intelligence approaches for predictive purposes. In addition to the artificial neural network and fuzzy logic approaches, an artificial neural network model based on fuzzy logic was also used, and the results were compared. Al-Habaibeh and Gindy [14] examined the number and type of sensors in milling to investigate tool conditions. The aim of this study is to facilitate design optimization and development by reducing the cost and time of the turning process of AISI 5140 steel alloy. In the study, the Taguchi experimental design was used and *AE* (Acoustic Emission), vibration, current and cutting force signals were collected and evaluated. Oraby and Hayhurst [15] employed non-linear regression analysis to develop models that can estimate tool wear/life in terms of force ratios, instead of using absolute values of force. During the turning of stainless steel, a sensor system was established with the help of neural networks to determine formation using cutting forces and *AE* signals [16]. Tool wear values were measured offline, and AE and power signals were measured on-line. It was observed that the *AE* signals were successful in tool wear monitoring, and the axial force increased with the increase of tool wear so that cutting forces could also be used. Zhang et al. [17] presented a sensor system that can predict tool wear and tool life, defined as a low-cost and effective manufacturing optimization method. The system includes vibration, shear force and power sensors. The results showed that the multi-sensor system is an effective method.

Ratava et al. [18] established a sensor system to examine the intermittent cutting situation that occurs during the turning of a flywheel, and to monitor the wear occurring in the cutting tool. The system is based on the use of acceleration sensors to estimate the tool deflection as a source of the actual cutting force. It was found that this method can be used to prevent catastrophic failure, and to prevent small breaks in the tool. Ambhore et al. [19] observed tool wear using the vibrations that occurred during the turning of an AISI 52100 material. Multiple regression models were used to show the relationship between tool vibrations and tool wear. Ong et al. [20] established a sensor system based on an artificial neural network and examined the effect of different levels of cutting speed, feed, depth of cut and machining time on surface roughness and tool wear. *V_B_* estimation was made using an artificial neural network. In addition to cutting parameters and machining time, tool wear can be successfully estimated with the contribution of the image processing method. Panda et al. [21] performed the monitoring of tool wear, surface roughness and vibration in the turning of AISI 52100 material. According to the results, cutting speed was the most effective parameter on *V_B_*, surface roughness and vibration. Bhuiyan et al. [22] introduced a method that separates the signal frequency that occurs during tool wear and plastic deformation using an *AE* sensor. As a result, it was revealed that the two cases showed variation in their frequency ranges. While tool wear frequency fluctuates in different stages of wear, *AE* signal amplitude increases with the increased tool wear and depth of cut. Based on the previous literature [13,14,15,16,17,18,19,20,21,22], it can be concluded that none of the previous studies utilized various sensors.

Azouzi and Guillot [23] have designed a system based on a sensor system and an artificial neural network, using force, vibration and temperature sensors. Successful predictions were carried out with an acceptable error rate in the study, wherein the dimensional deviation and surface finish were successfully estimated. Chen and Jen [24] developed a sensor system model based on cutting force and vibration measurements in milling. A sensor system based on an artificial neural network measurement was established, tool wear estimation was also performed, and network training and test performance were evaluated. Mali et al. [25] developed a sensor system based on force and vibration measurement in turning the Inconel 718 material. With this system, tool wear was monitored in real-time and the results were evaluated with variance analysis based on the Taguchi method. It has been observed that tool wear estimation with sensor systems can be obtained with higher accuracy. Duro et al. [26] conducted a study to understand the most accurate sensor region for monitoring the cutting process, and which sensor signal defines the process most accurately. They aimed to use different signal features related to the cutting process, and make an estimate by using a sensor system. Wu et al. [27] designed an information and prediction system based on a fuzzy logic extraction system using a sensor system. In this way, the amount of wear was estimated by a method based on multiple sensor systems, and *AE*, vibration and current sensors were used. Looking back at the summarized literature, it can be concluded that none of the previous studies monitored tool wear and surface roughness during the turning of AISI 5140 steel.

It is important to obtain desired surface roughness in machining operations, as it is the final process in the production of a part. In this manner, a variety of methods for measuring the surface roughness in order to minimize flatness, model surface topography and monitor with sensors exist. Pimenov et al. [28] reported a study on modeling flatness deviation while compensating for the torsional angle of the milling face. Quinsat and Tournier [29] proposed an in-situ measurement system for surface roughness to solve the problem of removing the workpiece material from the machine tool. Nieslony et al. [30] performed a study based on solving the turning of mould parts precisely for topographic inspection and surface roughness. Wojciechowski [31] carried out a study on the relationship between tool displacements and surface quality at the end milling process. Krolczyk [32] demonstrated the description of surface topography in dry and lubricated cutting conditions, and found that the roughness of the surface can be reduced by using lubrication. Li et al. [33,34] presented studies of on-machine surface measurement to increase accuracy and efficiency during machining operations. Shahabi and Ratnam [35] utilized machine vision for the monitoring of tool wear and surface roughness, and the effect of nose wear on surface quality was investigated as well. Rao et al. [36] presented a TCMS for a boring operation measuring surface roughness in addition to vibration and material removal rate, and an empirical relation was obtained for tool life and surface roughness. Bustillo et al. [37] used different artificial intelligence methods, namely multilayer perceptron, radial basis networks and random forest, for the prediction of surface wear during electro discharge machining, turning and grinding operations. Pimenov et al. [38] proposed an artificial intelligence-based prediction using teeth wear in a face milling operation. They compared different methods, namely random forest, multilayer perceptron, regression trees and radial-based functions, and random forest was found as the most accurate model. Kassim et al. [39] used tool conditions for the identification of surface texture, and they reported that TCMS can be accurately implemented in analyzing surface texture.

Maruda et al. [40] presented an analytical approach for the different lubrication conditions after a turning operation. They intended to find the minimum surface roughness value, and reported that oil retention leads to an increase in surface roughness. Sivaiah et al. [41] compared the effect of cryogenic coolant conditions with a minimum quantity of lubrication, wet machining and dry machining. According to the results, cryogenic machining seems the best option for surface integrity and tool wear. Priarone et al. [42] tested the performance of Cubic Boron Nitride (CBN) and Polycrystalline Diamond (PCD) cutting tools for tool life and surface integrity under cryogenic cooling and conventional cooling. Cryogenic cutting with PCD provided the best solution. Da Silva et al. [43] focused on surface integrity and tool life under different cooling conditions, and high-pressure coolant provided the best tool life. Said et al. [44] investigated the studies on using nano-cutting fluids comprehensively. Sarıkaya et al. [45] studied the effects of dry, conventional cooling and minimum quantity lubrication methods on tool wear and surface roughness. They obtained the optimum cutting conditions for the cutting parameters and lubrication conditions of the turning of Haynes 25. Sartori et al. [46] purpose to test the different minimum quantity lubrication strategies for better tool life and surface integrity using a solid lubricant. They found that new strategies gave increased tool life and surface integrity. Mia et al. [47] developed an approach to hard turning under different cooling conditions, and found that the minimum quantity of lubrication provided desirable machining conditions.

In machining, TCMS, which comprises sensors, data acquisition equipment, data processing software and measuring devices adapted to machine tools, is required for determining machinability. Sensors can monitor a mechanical system to detect the changes and transmit the information for further evaluation with computer software. From this point of view, it is seen that it has relevance to sensor system [3,13,14] and wear-roughness inspection [48,49] studies. Investigating the effects of cutting parameters via ANOVA provides information on the availability of each sensor’s data in the monitoring of response parameters, i.e., flank wear and SR. Beyond following a quality indicator, TCMS can make decisions with an artificial intelligence supporting system by making on-line deductions about the condition of the process.

The workpiece material AISI 5140 was chosen because of its wide range of utilizations in the automotive and energy industries. The alloy has a high-strength and -hardness structure that leads to excessive cutting forces and cutting vibrations, and finally make it difficult for machining. This situation arises from the high chrome content and resultant hard carbides. The studies on machining AISI 5140 steel in the open literature are limited. Furthermore, the use of multi sensor systems simultaneously to monitor machining outputs such as tool wear and surface roughness is not reported. On the machinability of AISI 5140, Grzesik [50] explored wear development in hard turning, and found abrasive, adhesive and fracture generation on ceramic tools. Ebrahimi and Moshksar [51,52] investigated the effect of cutting speed, feed rate and cutting force on flank wear during boring, and the influence of cutting speed, feed rate and hardness on tool life in the turning operation. Li et al. [53] performed a study based on different coating conditions, and found that TiN-coated tools demonstrated abrasive and adhesive wear, while TiAlN-coated tools showed adhesive, diffusive and oxidation wear. Zhang et al. [54] examined an experimental study for sintered tools to obtain wear mechanisms and optimum cutting conditions during turning. According to the results, cermet tools offered a better performance than cemented carbide tools. Bican et al. [55] researched the influence of boriding, quenching and tempering after boriding on the wear development of AISI 5140, and they found that quenching and tempering after boriding reduces tool life. The investigation of the machining of AISI 5140 only concerned grinding; for example, Huang et al. [56] examined the effect of lubrication, Köklü [57] researched the effect of different-angled grooved grinding wheels on AISI 5140, and Grzesik and Wanat investigated the surface roughness under different feed rates. For the optimal cutting conditions of AISI 5140, Kahraman [58] performed an optimization approach in the turning of AISI 5140 steel for better surface roughness. Aslan [59] also proposed an optimization and analysis for minimum flank wear, cutting forces and vibration. As can be seen, limited research exists in the open literature about AISI 5140 carbon steel, and none of it handles the turning of this material based on a multi-sensor system. One of the main objectives of this study is to fill this gap.

ANOVA is a widely used statistical technique in many fields of research. It determines and quantifies the effective experimental factors (i.e., cutting parameters) affecting the measured outcome of an experiment (i.e., machining quality characteristics). Accordingly, the levels of effectiveness of the parameters with various statistical values are expressed numerically. A large number of researchers employed statistical analysis to study the various machining parameters of a wide range of materials under different cutting conditions, which reflects the success of this approach [3,60,61,62,63,64].

Having more parameters and variables, turning is applied in industrial studies as the most common manufacturing method. To eliminate failures and maximize productivity, sensor-based systems have been used in the past, as was mentioned before. Only one or a group of these sensors were compared and evaluated, but the success of all in the detection of several results cannot be seen in the literature. The summarized review indicates that no previous study seems to pursue an investigation of the influence of cutting speed, feed rate and depth of cut on *V_B_* and *Ra*, based on multi-sensorial data, namely cutting force, vibration, *AE*, temperature and motor current. The investigation of cutting parameters for all the cutting variables provides a comprehensive guideline for scientists and manufacturers to obtain high quality products, and makes it easy to evaluate the resultant cutting variables during turning of AISI 5140.

To fill this gap, this paper deals with five different sensors and vital quality characteristics; specifically, *V_B_* and *Ra* were evaluated with ANOVA. The experimental plan involved using the Taguchi method. Besides, RSM-based optimization was applied to *V_B_* and *Ra* and sensorial data. Three levels of cutting speed, feed rate and depth of cut were used to compose the experimental study during the dry turning of an AISI 5140 material. Cutting force, vibration, *AE*, temperature and motor current measurements were performed as on-line during machining, while *V_B_* and *Ra* were measured as off-line when the tool machine was stopped. A statistical, and subsequently an optimization, approach with various measurement devices provides broad perspectives, and provides practical recommendations for the turning of AISI 5140 steel.

## 2. Materials and Methods

Considering the studies in the literature and using the existing infrastructure, a pre-experiment and then an experimental study was carried out. In this study, a cylindrical workpiece of AISI 5140 steel was mounted onto a universal lathe and peripheral turning was performed in various machining parameters. The material used is called AISI 5140, medium carbon alloy steel. It had a cylindrical geometry and was cut into shapes of 75 mm diameter and 500 mm length. The chemical composition of the workpiece material is given in Table 1. Experiments were carried out on the lathe (De Lorenzo S547-8899-Italy) under dry cutting conditions. The Physical Vapor Deposition (PVD)-coated carbide cutting tool’s geometry (WCMT 06T308-P25-Achteck-China) and 90° approaching angle were chosen.

The cutting tool nomenclature is demonstrated in Table 2. A new workpiece material was used for each experiment and six pass chips were removed with an insert. Derived from the manufacturer’s handbook and the machine tool’s operation range, the cutting parameters were selected as three cutting speeds (150, 200 and 300 m/min), three feed rates (0.06, 0.12 and 0.24 mm/rev) and three depths of cut (1, 1.5 and 2 mm). The cutting parameters and factor levels are presented in Table 3. In this paper, in order to decide the number of experiments, a Taguchi L_9_ orthogonal array was used which is demonstrated in Table 4. Taguchi is a method proposed for experimental design [65] and it has set various objectives for clarifying the definition of quality engineering. The Taguchi approach aims to reduce costs while improving quality. For this purpose, it uses orthogonal arrays and S/N ratio. Orthogonal arrays aim to result in a study with a minimal number of runs in order to help reduce machining costs by putting forward an experimental design, while the S/N ratio provides a high quality result by eliminating the noise factors.

**Figure 1:**
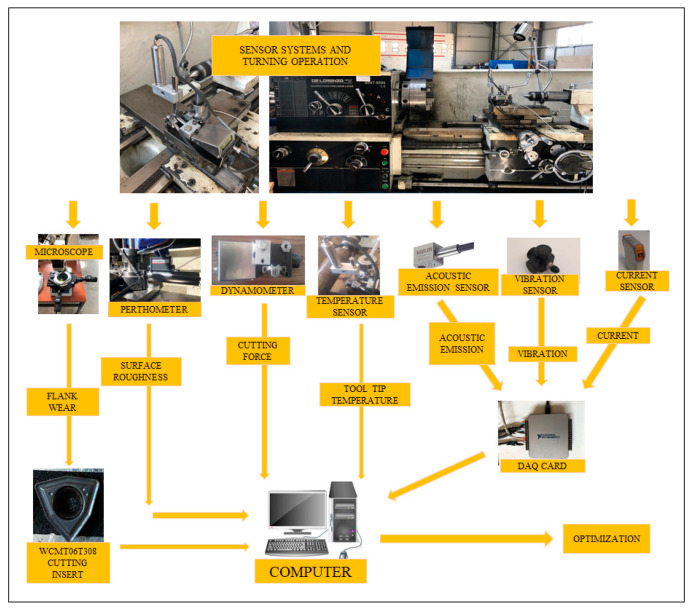
Experimental setup for turning.

The experimental setup includes the machine tool, the measuring devices, sensors, data acquisition units and a computer. The experimental setup is shown in Figure 1. The measurement devices and sensors were calibrated to better interpret the results of the experiment and reduce the error rate when making analyses. In this sense, to make a more accurate comparison with past studies and to contribute to future studies, the selection of materials and methods is of great importance. In the experimental study, a turning dynamometer (TelC DKM 2000-Germany) was used to measure the cutting force. The dynamometer’s body also acts as a tool holder, and can be attached to the carriage. At the top of the dynamometer there is an apparatus that allows the temperature sensor to be mounted. In this way, the sensor attached to the tool holder moving in the feed axis measures the temperature at the tool tip. The temperature measurement sensor (TeLC-Germany) used was designed to measure the temperature at the cutting tool-tip and can detect the tool tip temperature with the InGaAs radiation measurement method. This sensor can be mounted on the dynamometer and adjusted within a 100 mm distance from the cutting tool tip, as determined in the manufacturers handbook. Both dynamometer and temperature sensors can be directly connected to the computer, and using the software (XKM2000-Germany), data tracking, processing and recording can be performed.

In this study, an *AE* sensor (Kistler 8152B111-Switzerland) on the machines was used to measure signals from emission sources in the range of 50–400 kHz. The sensor was mounted on the dynamometer so that it is positioned as close as possible to the cutting tool. An accelerometer (Kistler 8692C50-Switzerland) was mounted on the machine parts by means of the magnetic clamp at its base. A motor current sensor (Weidmüller WAS2 CMA 5/10A-Germany) was used to transmit the signals to the computer via a data acquisition card (National Instruments USB-6003-USA). One of the cables that transmits current to one of the main shafts that transfers power to the machine tool is passed through the channels of the sensor. Both *AE* and the accelerometer transmit the collected data, via a data acquisition card, to the computer. The data acquisition card is capable of connecting to the computer easily, and transferring data via USB connection. For each experiment, six specimens were machined using one tool tip under the same cutting conditions to investigate tool wear and surface roughness. After the completion of two passes, the operation was stopped and *V_B_* and *Ra* were measured three times for every experiment as off-line. *Ra* was measured using a Mahr M1 Perthometer roughness tester (Germany). *V_B_* was measured using a Mitutoyo TM-A505B 176-820A microscope (Japan). A toolmakers microscope allowed the measuring of flank wear in a non-contact way via micrometers positioned at two sides of the device. The cursor can be arranged with the help of these micrometers, and it can be moved on the flank face of the cutting tool. The distance from the edge can be measured in two directions to determine the depth and width of wear. Surface roughness was measured with the portable probe and directly obtained from the surface of the workpiece on the machine tool. Measurements were carried out of the circular face of the workpiece and it was performed at equal intervals (120°). *V_B_* and *Ra* measurements were repeated three times for reliable evaluation. The average values of all the measurements collected during the experiments were determined.

## 3. Results and Discussion

In today’s technology, it is an easy process to collect data from a single sensor with existing equipment and data acquisition devices, but the process may be insufficient at the point of monitoring. Although the increase in the number of sensors makes it possible to collect more data about the process, on the other hand, it can cause misinterpretations due to the complexity of the data obtained. The presence of many more or less effective parameters and variables in a quality indicator in machining produces a nonlinear relationship between the parameter to be examined and the variables, and causes the mentioned complexity. To prevent this complexity, one should eliminate the noise in the signals with the help of artificial intelligence methods, and verify by comparing the information. In this context, ANOVA- and RSM-based approaches were used to measure the capability of each sensor, which is generally preferred for obtaining information about tool and workpiece condition. Cutting force, cutting temperature, vibration, *AE* and motor current were measured on-line during machining and at the end of the process; *V_B_* and *Ra* measurements were also performed.

### 3.1. Experimental Results and ANOVA

The experimental design and results are laid out in Table 4, representing the responses and sensorial data separately. The factors and levels represent the experimental design, namely the Taguchi L_9_ orthogonal array, while the response parameters and sensorial data show the experimental results. In Table 4, *V_B_* and *Ra* represent response parameters, and cutting force, vibration, *AE*, temperature and motor current define the sensorial data collected and measured in the experiments. Table 5 shows ANOVA results for the response parameters, namely *V_B_* and *Ra*, and Table 6 shows ANOVA results for sensor data, namely cutting force, *AE*, temperature, vibration and motor current.

ANOVA is preferred in many fields and engineering applications, and in the field of machining that this study covered. The purpose of ANOVA is to determine which design parameters are effective on the quality characteristics, namely manufacturing cost, tool life and workpiece quality. Accordingly, the degrees of effectiveness of the parameters with various statistical values are expressed numerically. The change of the variable parameters of a quality characteristic causes total variability in the quality characteristic. The sum of squares of the quality characteristics are calculated below, with the mean value and the difference between the results of each experiment, and ultimately, the result for each experiment is calculated. While each design parameter has a certain effect on this total, the remaining result from the sum of these effects gives the error. By dividing the sum of the squares belonging to the parameters by the sum of squares, the amount produced as a percentage of that parameter is calculated. Another statistical parameter is the value expressed as mean squares, which is calculated by dividing the sum of squares for each design parameter and error by the degree of freedom. Then, the value of the average square of the parameter is divided by the mean squares error value to calculate the result known as the F-value. A high *F*-value indicates that the degree of effectiveness of the parameter is high. Another important feature is the *P*-value, and accordingly, statistical analysis is carried out in a confidence interval, which is usually 95%. As a result, if the calculated value is lower than 5%, this is because the parameter is important. The statistical results obtained with ANOVA reveal the effect of the design parameters within a certain range, namely the experimental plan. This is why the generally accepted effects have undergone some change. The above-mentioned statistical results give consistent results in a good analysis.

### 3.2. Analysis of Surface Roughness and Flank Wear

Table 5 demonstrates that cutting speed (46.1%) is the parameter that is most effective on *V_B_*, followed by the depth of cut (45.6%) and feed rate (0.8%), which made no significant contribution to wear. Increasing cutting speed enhances the coefficient of friction and cutting temperatures, which expedites the wear rate of the cutting tool. Debnath et al. stated that cutting speed and depth of cut have a great influence on tool wear [66]. Since the increase in the depth of cut raises the chip removal rate, it affects the temperature substantially, and also affects the wear via the effect of the cutting speed [67]. The effect of the depth of cut on *V_B_* has been investigated [22], and it has been revealed that with the change in *AE* signals, the increase in depth of cut affects *V_B_* more than cutting speed and feed. Yu et al. found that the cutting temperature, which had a huge impact on wear, reached the highest level with an increase in the depth of cut [68]. Mia et al. found that the most effective parameter on *V_B_* was the depth of cut [69]. It was found that the increased depth of cut leads to a high amount of chip removal, and the cutting tool is subjected to more restrictive force, causing the workpiece to shear. However, the thermal stresses in the tool increase the amount of plastic deformation and trigger tool wear.

In Table 5, *Ra* analysis results are also demonstrated, which outline the effects of cutting speed, feed rate and depth of cut separately. Feed rate seems to be the dominant parameter (62.3%) effecting surface roughness, followed by the depth of cut (20.7%). It has been found with theoretical models that the most effective parameters for surface roughness are tool nose radius and feed rate. Roughness is essentially a term that expresses the irregularity of a surface, and it is obtained by calculating the depth of the peaks and hollows in the surface as a result of the advance of the radius. The reason why these two parameters are effective can be explained in this way. In the past studies, it has been observed that feed rate generally affects the surface roughness. For example, Khanna et al. [70] and Asiltürk and Akkus, [71] showed that feed rate is more effective on *Ra* compared to cutting speed and depth of cut. Debnath et al. [66] experimentally found that the parameter that is most effective on surface roughness is feed rate. According to the statistical results, the percent contribution (PC), F-value and P-value seem compatible with each other, which proves the reliability of analysis for both *V_B_* and *Ra*. The statistically reliable results of tool wear and surface roughness provide the promise, in the context of machining operations, of obtaining optimal solutions, as these outputs are accepted as the ultimate aim during manufacturing.

**Figure 2:**
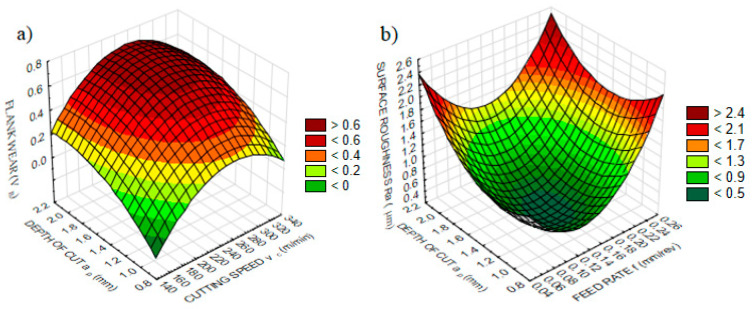
The variation of measured variables according to effective parameters: (**a**) flank wear and (**b**) surface roughness.

**Figure 3:**
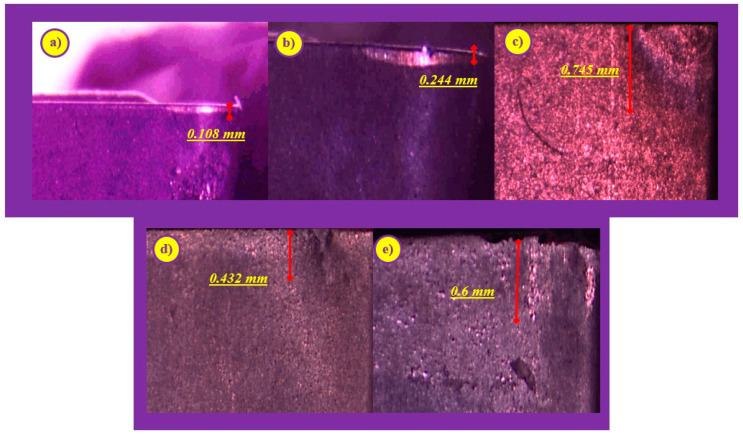
Flank Wear Developments on Cutting Tools for: (**a**) *v_c_* = 150 m/min, *f* = 0.06 mm/rev, *a_p_* = 1 mm; (**b**) *v_c_* = 150 m/min, *f* = 0.24 mm/rev, *a_p_* = 2 mm; (**c**) *v_c_* = 200 m/min, *f* = 0.12 mm/rev, *a_p_* = 2 mm; (**d**) *v_c_* = 330 m/min, *f* = 0.06 mm/rev; *a_p_* = 2 mm; (**e**) *v_c_* = 330 m/min, *f* = 0.24 mm/rev, *a_p_* = 1.5 mm.

According to ANOVA results, depth of cut and cutting speed influence *V_B_*, while feed rate and depth of cut seem effective on *Ra*, which is represented in Figure 2 on three-dimensional graphs. *V_B_* is directly proportional to the depth of cut for all cutting speed values (Figure 2a), which can be understood as an effect of the larger chip cross-section that aggravates chip removal [68]. Furthermore, the cutting temperature rises with increasing depths of cut, and temperature is accepted as the major trigger of tool wear [72]. On the other hand, *V_B_* improves up to a certain value with the increasing cutting speed, and then shows a slightly decreasing trend for all depth of cut values. It is expected that with the increase in cutting speed, *V_B_* accelerates as a result of the effect of cutting speed on the friction between the tool and workpiece, and cutting temperature [66]. In Figure 3, *V_B_* developments can be seen for different cutting conditions. Higher values of cutting speed and depth of cut are observed according to alterations in tool wear. The maximum values of depth of cut or cutting speed increase *V_B_* excessively, according to our results.

The most effective factors on surface roughness are feed rate and tool tip radius [73]. The tool tip radius was kept constant in this paper. Surface roughness increases with the increasing feed rate because of the enhanced tendency toward waviness [74]. Depth of cut is assigned according to operation type, and plays a significant role in determining the cutting tool and workpiece contact [75]. Therefore, the depth of cut reflects the preset contact conditions between tool and workpiece, and the desired surface roughness with the help of feed rate and tool tip radius. According to the three-dimensional graph, *Ra* increases with the increase in the feed rate, whereas the increase in depth of cut increases *Ra*, as shown in Figure 2b. With three-dimensional graphs, the observation of the effective parameters is easier compared to two-dimensional graphs, and further analysis can be carried out for optimization.

### 3.3. Analysis of Sensorial Data

The effects of cutting parameters on sensorial data, namely cutting force, *AE*, cutting temperature, vibration and motor current, were statistically analyzed via ANOVA, as shown in Table 5. The literature review indicated that ANOVA-based statistical analysis is generally carried out for cutting force. One of the purposes of this work was to investigate several sensorial datasets. Since the sensor signals are representative of a successful and well-informed machining operation, the determination of these variables provides noteworthy information about the tool wear, surface roughness and the general progress of machining.

The cutting force analysis results show that feed rate is the most effective parameter (69.1%), followed by the depth of cut (28%). The statistical results of cutting force appear to be compatible with both theoretical results and past studies. If the situation is analyzed in terms of theoretical models, it can be seen that the depth of cut and the feed have an impact on cutting force [69]. The basis of this is that the cutting force is theoretically formed as a result of the joint effect of the specific cutting force and the chip cross-section, and the chip cross-section is dependent on the depth of cut and feed rate. It is also observed that the cutting force is raised as the increase in these parameters enhances the chip cross-section. Considering the studies in the literature, it was found that progress is the most effective parameter on the tangential cutting force [3]. Dimla stated that the feed and depth of cut have effects on the cutting force, and this is due to tool wear and a lack of homogeneity in the workpiece [76].

It can be observed in the results that the parameter that most affected *AE* is the cutting speed (46.1%). *AE* is a phenomenon that increases as a result of factors such as deformation, collision, friction and breakage [22]. The conclusion that can be reached is that the increase in cutting speed causes greater changes in these factors. The increase in cutting speed not only increases the deformation in the workpiece and the friction between the tool and the workpiece, but also affects the chip evacuation and its orientation [77,78]. According to Dolinsek and Kopac [79], tool wear significantly affects the *AE* signal structure. On the other hand, with developing tool wear, *AE* signals demonstrates enhancing tendencies [22]. Kuntoglu and Saglam reported that cutting speed is the most effective parameter with regard to *AE*, according to ANOVA results [3].

The high speed applied to the cutting tool or workpiece during chip removal, and the relative movement of the workpiece with the cutting tool, creates a high temperature and pressure in the cutting zone [80,81]. In processes determined by various cutting parameters, high plastic deformation occurs in the region where the cutting tool and workpiece come into contact [1]. The high friction caused by the pressure and temperature that provide chip formation, and the force of the cutting tool in sliding on the workpiece, create time-dependent deformations in the cutting tool [2]. In obtaining a surface and product with certain features and quality, deterioration in the cutting tool is inevitable. As a result, cutting temperatures are the primary factor in developing cutting tool wear. This situation is triggered principally by high cutting speeds. In Table 6, cutting temperature analysis is demonstrated, and cutting speed is the most important factor, with a contribution rate of 89.9%.

In general terms, vibration can be described as an oscillation around an equilibrium point, which manifests a disruption in the contact area between the cutting tool and workpiece [82]. The theoretically determined tool geometry present during the relative movement of the cutting tool and the workpiece causes undesired results, such as undesired chip shape, deterioration of the workpiece surface form, and the loss of tolerance and unexpected wear structures [83]. It is hard to compare vibration results with past studies because of the lack of statistical analyses and the incomprehensible mechanism of vibration. In [84], the authors reported that with the increase in cutting speed, higher vibration amplitudes can be obtained. In the context of this study, the feed rate was found to be the dominant factor influencing vibration, and it can be interpreted as to be a trigger of vibration because of its effect on chip formation. The important part of this result is that it illustrates the dominant parameter in vibration, which is feed rate (41.1%).

In machining, the motor current drawn by the main motor and the power consumed vary depending on the wear and changing cutting forces occurring in the cutting tool during cutting. The wearing tool starts to lose its cutting ability gradually due to the deterioration of the cutting geometry. This situation causes the cutting forces to increase, and the machine to draw more motor current [85]. The same as the vibration results, statistical analysis- and current-based research is hard to find. The investigation is limited to finding the parameters that are most effective with regard to current because of the low contribution rate (74.6%,). According to the results, the depth of cut has more effect on current (39%), followed by feed rate (23.3%) and cutting speed (12.2%), respectively.

**Figure 4:**
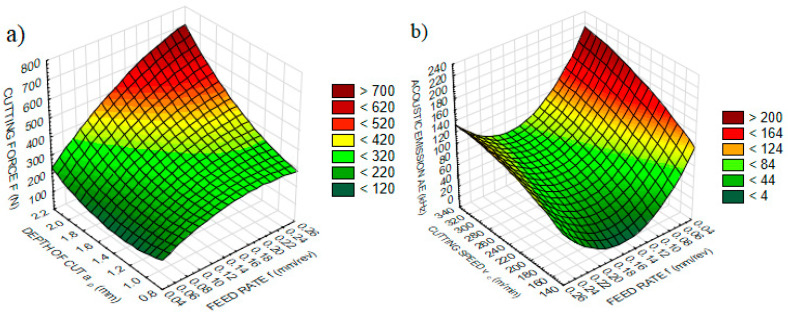

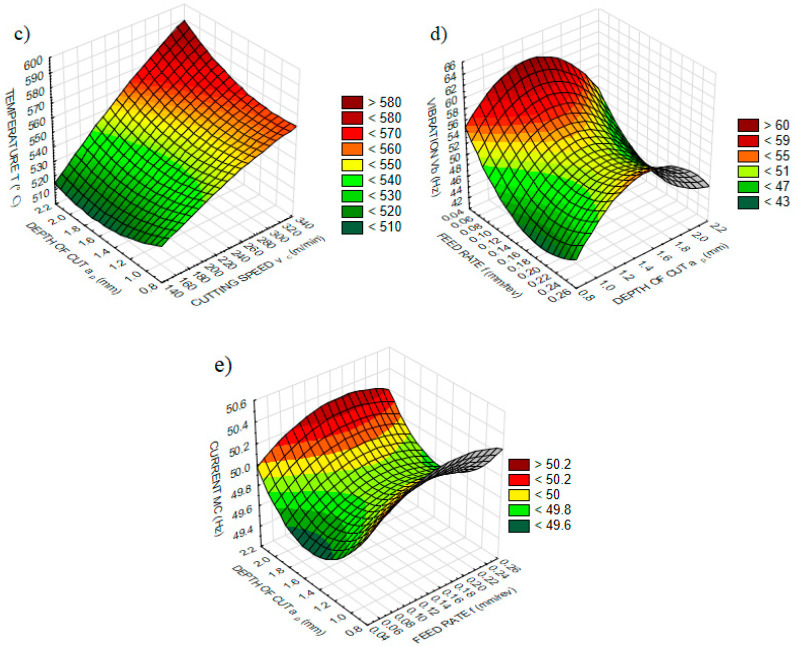
The variation of sensorial data according to effective parameters: (**a**) cutting force *F* (N), (**b**) acoustic emission *AE* (kHz), (**c**) temperature *T* (°C), (**d**) vibration *Vb* (Hz), (**e**) current *MC* (Hz).

The changes in feed rate, cutting speed or depth of cut, and the result of these changes, fluctuates the process parameters. Thus, it can be inferred that if the amount of change in these parameters can be determined, then a relationship can be established between them. Figure 4 represents the effects of influential parameters obtained from ANOVA, and the changes of sensorial data values, in three-dimensional graphs.

The basic and most influential parameters on cutting force are accepted as being feed rate, depth of cut and specific cutting force [75]. Increasing the feed rate and depth of cut enhances the cutting force as a result of increasing the cutting area [3]. The cutting force indicates an accelerating behavior, and reaches the maximum value with the combined effect of the highest feed rate and depth of cut (Figure 4a).

*AE* is generated from contact areas between tool, workpiece and chip as a result of different types of events, such as tool wear or breakage, deformation of the tool and workpiece, chip removal, collision or breakage [86]. One of the important factors triggering the *AE* is tool wear [79]. The results reported in Figure 2a show that increasing the cutting speed enhances the tool wear, and consequently increases tool–workpiece contact area, and thus the coefficient of friction is obtained. As a result, *AE* demonstrates an increasing trend along with increasing cutting speed (Figure 4b).

Basically, tool wear is accepted as a result of the temperature-dependent mechanism [87]. Under high pressure, cutting tool, workpiece and chip contacts generate temperature as a consequence of friction force. A relationship between friction force, cutting temperature and tool wear is established which fits to theoretical assumptions. Induced tool wear and temperatures are results of high speeds, and according to [80,88], enhancing cutting speed increases the temperature. It is observed that increasing the cutting speed and the depth of cut accelerates temperature (Figure 4c). Enhancing the depth of cut also plays a similar role, and has an important effect on enhancing both tool wear and temperature.

Being a complex phenomenon, vibration appears as an unstable event that arises from the friction-dependent mechanisms between cutting tool, workpiece and chip [83]. It can be observed that any change in the stability of the cutting mechanism leads to undesirable vibrations [89]. There is a need to identify the distorting factors in vibration, which can affect the workpiece quality. Vibration improves with reductions of the feed rate, but shows no significant change with the depth of cut (Figure 4d). It can be concluded that the increase in vibration manifests in surface roughness by restraining and decreasing the *Ra* value at lower feed rates, as shown in Figure 2b for all depth of cut values.

Since there is a lack of studies on motor current, inquiring about the process condition via comparisons between past studies becomes difficult. Furthermore, as these studies approach the cutting area from a distance, they are disadvantaged in collecting reliable data via the motor current. Apparently, the depth of cut influences motor current, which decreases to a value and then shows an increasing trend (Figure 4e).

### 3.4. Optimization of Process Variables in Response to Surface Methodology

RSM is a method of modeling parameters defined as independent variables so as to optimize the results described as dependent variables. RSM is used to understand the behavior of the machine, and the optimization of parameters can be obtained using a statistical approach [90]. One of the main advantages of this method is its inadequacy in optimizing multiple parameters simultaneously. In turning, some researchers preferred to use this method for optimization and modeling [91,92]. In this manner, during the building phase of the model, each parameter can be assigned to associated objective functions for minimizing or maximizing. Consequently, the model provides an optimal solution for all parameters embodied in the model individually and totally. In the creating process, the weight and importance values were selected as “1” so as to observe the efficiency of each parameter. Since each parameter is ideally held at its minimum level, the goal was selected as minimizing during the setup in Minitab 16. The optimal solutions of multiple results have great importance in saving the quality, improving the efficiency and accuracy and decreasing wasted material and time, especially for big data, as demonstrated in the following paragraphs.

**Figure 5:**
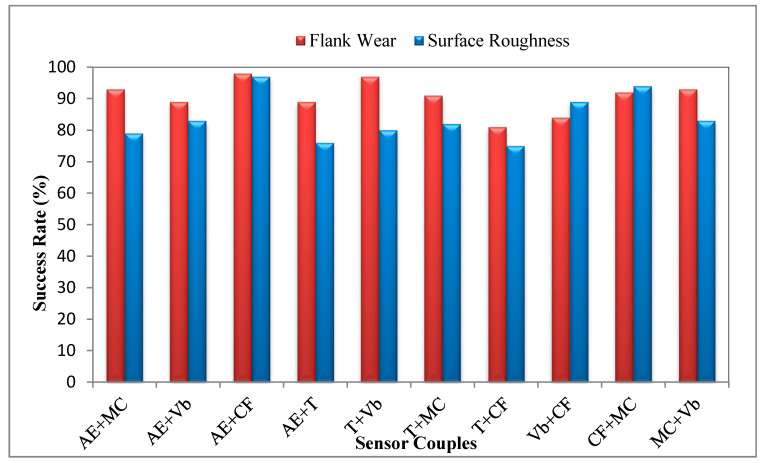
Success Rate of Sensor Couples for Flank Wear and Surface Roughness.

**Figure 6:**
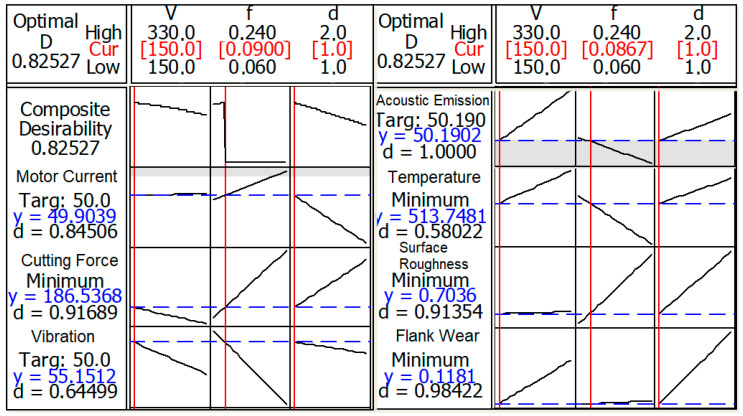
RSM-based optimization of variables.

The obtained values from the RSM software for each parameter, and their desirability, are shown in Table 7. Except for the temperature and vibration, the other parameters can be optimized in satisfying desirability values. For the composite or total desirability, an 82.5% success rate was found, which is highly satisfying and promising for future studies, especially considering the complex structure of the turning operation. To compare and support Table 7, sensor couples were determined for the optimization of *V_B_* and *Ra* separately in Figure 5. According to the results, the cutting force and *AE* signals were found to be the best sensor couples for the optimization of *V_B_* (98%) and *Ra* (97%), respectively. The table gives optimal values of composite desirability and the variables is demonstrated in Figure 6. The optimum parameters were found to be *v_c_* = 150 m/min, *f* = 0.09 mm/rev and *a_p_* = 1 mm for multiple optimizations. For verification of the optimum parameters, the obtained results were tested under these cutting conditions in an additional experiment, and are explained in detail in the next section.

### 3.5. Confirmation Experiment

In order to verify the accuracy of the developed model, a confirmation experiment was carried out. A comparison of the additional experiment and predicted results was performed. According to the result, the percentage error was found to be within 10% (Table 8). As a result, the developed model based on RSM offers accurate results for several combinations of cutting speed, feed rate and depth of cut in predicting too many variables. Considering the dynamic interaction between these variables in the complex nature of the turning operation, obtaining experimental values within the range of 91.2–99.9% indicates the success of the approach, and its suitability for future studies. With confirmation, the repeatability and reliability can be tested for future studies, and for similar applications with different materials.

Other verification experiments are listed in Table 9 considering the success rates of sensor couples in Figure 5. According to that figure, based on RSM, the most effective sensor signal couples for the optimization of *V_B_* and *Ra* seem to be acoustic emission and cutting force. Given this solution, the cutting conditions should be *v_c_* = 150 m/min, *f* = 0.06 mm/rev and *a_p_* = 1 mm for both *V_B_* and *Ra* (the composite desirability is 0.97). After that, the temperature–vibration and motor current–cutting force sensor signals were determined to be effective in predicting *V_B_* and *Ra*, respectively. This time, the optimal solutions were determined to be *v_c_* = 150 m/min, *f* = 0.24 mm/rev and *a_p_* = 1 for *V_B_*, and *v_c_* = 330 m/min, *f* = 0.06 mm/rev and *a_p_* = 1 for *Ra*, with composite desirability values of 0.96 and 0.94, respectively. The tabulated results demonstrate that the prediction models are accurate and are in good agreement with experimental values.

## 4. Conclusions

This study shows the statistical analysis and optimization of experimental results for the turning of AISI 5140 steel. In this context, a comprehensive data acquisition process has been carried out in order to collect five different sensorial datasets (cutting force, acoustic emission, tool tip temperature, tool vibration and motor current), additionally to surface roughness (*Ra*) and tool wear (*V_B_*) measurements, for the first time in the open literature. Different from the previous studies, five different sensors were integrated into a lathe for on-line data acquisition on the whole, in addition to off-line measurements of surface roughness and tool wear. ANOVA-based statistical analysis and RSM-based optimization provided a series of numerical results, which can be a further supportive data source for TCMS- and artificial intelligence-based systems. TCMS-based monitoring of the turning operation provided accurate analysis and optimization results for *Ra* and *V_B_* via multi-sensorial data. The results will be practically useful for future studies, considering the extensive data derived from various sensors in the turning of AISI 5140 steel. The following conclusions are drawn from this paper:

- Feed rate has effects on *Ra* (62.3%), vibration (41.1%) and cutting force (69.1%), while cutting speed dominates *V_B_* (46.1%), *AE* (46.1%) and cutting temperatures (89.9%). The depth of cut has no important effect on sensorial data and response parameters, except for the motor current (39%), according to ANOVA;
- ANOVA also demonstrates the reliability of the analysis result, and cutting force (97.8%) is the most reliable sensor data, with *AE* (95.7%). Temperature (92.9%), vibration (81.3%) and motor current (74.6%) follow them, respectively;
- RSM-based optimization provides optimum cutting conditions for desirable experimental results, in order to obtain the best values simultaneously with high composite desirability (82.5%);
- The recommended optimum cutting conditions (*v_c_* = 150 m/min, *f* = 0.09 mm/rev, *a_p_* = 1 mm) were confirmed via additional experiments, with error rates (max. 9.8%) that are acceptable for this many variables.
- The comparison of the success rate of sensor couples for the optimization of *V_B_* (98%) and *Ra* (97%) demonstrated that dynamometer and *AE* sensors can be considered the most suitable sensors.

## Figures and Tables

**Table 1:** The chemical composition of AISI 5140 carbon steel.

Element	C	Mn	Si	Cr	Ni	Mo	V	S	Cu	P
%	0.45	0.7	0.28	0.85	0.14	0.05	0.029	0.065	0.01	0.02

**Table 2:** Cutting tool nomenclature.

W	C	M	T	06	T3	08	P25
Insert Shape	Clearance Angle	Tolerances	Chip Breaker	Length of Cutting Edge	Thickness of Cutting Edge	Corner Radius of Cutting Edge	Grade of Cutting Edge

**Table 3:** Cutting parameters and factor levels.

Symbol	Parameters	Level 1	Level 2	Level 3
*v_c_*	Cutting Speed *v_c_* (m/min)	150	200	330
*f*	Feed Rate *f* (mm/rev)	0.06	0.12	0.24
*a_p_*	Depth of Cut *a_p_* (mm)	1	1.5	2

**Table 4:** The experimental design and results.

Experiment Number	Factors and Levels			Response Parameters		Sensorial Data				
	Cutting Speed *v_c_* (m/min)	Feed Rate *f* (mm/rev)	Depth of Cut *a_p_* (mm)	Flank Wear *V_B_* (mm)	Surface Roughness *Ra* (µm)	Cutting Force *F* (N)	Vibration *Vb* (Hz)	Acoustic Emission *AE* (kHz)	Temperature *T* (°C)	Motor Current *MC* (Hz)
1	1	1	1	0.108	0.75	147.44	53.3	51.06	526.53	50
2	1	2	2	0.17	0.769	311.95	51.04	50.18	515.19	49.86
3	1	3	3	0.244	2.01	617.84	50.31	61.65	510.09	49.93
4	2	1	2	0.429	0.912	191.62	64.51	149	526.64	49.38
5	2	2	3	0.745	0.715	370.68	49.95	51.86	545.42	50.03
6	2	3	1	0.202	1.611	326.68	49.98	53.86	530.78	50
7	3	1	3	0.432	1.775	245.71	50.72	182.72	574.66	49.92
8	3	2	1	0.214	0.58	259.06	50.09	75.7	560.74	49.99
9	3	3	2	0.6	1.15	367	49.94	125.99	567.89	49.95

**Table 5:** Analysis of Variance results for response parameters, namely flank wear, surface roughness.

Cutting Parameters	Degree of Freedom	Sum of Squares	Mean Square	F Value	*p*-Value	Percent Contribution (%)
*FLANK WEAR V_B_* * *(mm)*						
Cutting Speed	2	112.939	56.469	6.26	0.138	46.1
Feed Rate	2	2.111	1.056	0.12	0.895	0.8
Depth of Cut	2	111.632	55.816	6.19	0.139	45.6
Error	2	18.037	9.019			7.4
Total	8	244.719				92.6
*SURFACE ROUGHNESS Ra* * *(µm)*						
Cutting Speed	2	0.205	0.1025	0.01	0.99	0.1
Feed Rate	2	76.142	38.0709	3.72	0.212	62.3
Depth of Cut	2	25.274	12.6368	1.24	0.447	20.7
Error	2	20.464	10.2319			16.7
Total	8	122.084				83.3

* R^2^ values for *V_B_* = 92.6%, *Ra* = 83.3%.

**Table 6:** ANOVA results for sensorial data, namely cutting force, AE, temperature, vibration and motor current.

Cutting Parameters	Degree of Freedom	Sum of Squares	Mean Square	F Value	*p*-Value	Percent Contribution (%)
*CUTTING FORCE F* * *(N)*						
Cutting Speed	2	0.666	0.3332	0.29	0.773	0.6
Feed Rate	2	71.777	35.8886	31.63	0.031	69.1
Depth of Cut	2	29.126	14.5632	12.83	0.072	28
Error	2	2.269	1.1347			2.2
Total	8	103.839				97.8
*ACOUSTIC EMISSION AE* * *(kHz)*						
Cutting Speed	2	73.377	36.689	10.8	0.085	46.1
Feed Rate	2	48.848	24.424	7.19	0.122	30.7
Depth of Cut	2	29.941	14.971	4.41	0.185	18.8
Error	2	6.793	3.397			4.3
Total	8	158.960				95.7
*TEMPERATURE T* * *(°C)*						
Cutting Speed	2	1.0132	0.5065	12.75	0.073	89.9
Feed Rate	2	0.0168	0.0083	0.21	0.825	1.4
Depth of Cut	2	0.0172	0.0085	0.22	0.822	1.5
Error	2	0.0794	0.0397			7.1
Total	8	1.1266				92.9
*VIBRATION Vb* * *(Hz)*						
Cutting Speed	2	0.7469	0.3735	0.96	0.511	17.8
Feed Rate	2	1.7206	0.8603	2.20	0.312	41.1
Depth of Cut	2	0.9272	0.4636	1.19	0.458	22.1
Error	2	0.7820	0.3910			18.7
Total	8	4.1767				81.3
*MOTOR CURRENT MC* * *(Hz)*						
Cutting Speed	2	0.001201	0.00060	0.48	0.674	12.2
Feed Rate	2	0.002286	0.00114	0.92	0.521	23.3
Depth of Cut	2	0.003824	0.00191	1.54	0.394	39
Error	2	0.002487	0.00124			25.4
Total	8	0.009797				74.6

* R^2^ values for *F* = 97.8%, *AE* = 95.7%, *T* = 92.9%, *Vb* = 81.3%, *MC* = 74.6%.

**Table 7:** Predicted responses with response surface methodology and desirability coefficients.

Parameter	Value	Desirability	Uncertainty
*Flank Wear*	0.118 mm	0.98	0.02
*Surface Roughness*	0.704 µm	0.91	0.09
*Cutting Force*	186.53 N	0.91	0.09
*Temperature*	513.74 °C	0.58	0.42
*Acoustic Emission*	50.19 kHz	0.99	0.01
*Vibration*	55.15 Hz	0.99	0.01
*Motor Current*	49.90 Hz	0.84	0.16
*Composite (Total)*	-	0.825	0.175

**Table 8:** Comparison of experimental and predicted results for all variables.

Experimental Result	Predicted Value	Experimental Value	Accuracy	Uncertainty
*Flank Wear*	0.118 mm	0.126 mm	93.7%	6.3%
*Surface Roughness*	0.704 µm	0.748 µm	94.2%	5.8%
*Cutting Force*	186.53 N	169.86 N	91.2%	9.8%
*Temperature*	513.74 °C	524.41 °C	97.9%	2.1%
*Acoustic Emission*	50.19 kHz	51.26 kHz	98%	2%
*Vibration*	55.15 Hz	52.87 Hz	95.7%	4.3%
*Motor Current*	49.90 Hz	50.01 Hz	99.9%	0.1%

**Table 9:** Comparison of experimental and predicted results for sensor couples.

**Experimental Result 1**	**Predicted Value**	**Experimental Value**	**Accuracy**	**Uncertainty**
*Surface Roughness*	0.618 µm	0.75 µm	82.4%	17.6%
*Acoustic Emission*	55.507 kHz	51.06 kHz	92%	8%
*Cutting Force*	151.78 N	147.44 N	97.2%	2.8%
**Experimental Result 2**	**Predicted Value**	**Experimental Value**	**Accuracy**	**Uncertainty**
*Flank Wear*	0.116 mm	0.108 mm	92.6%	7.4%
*Acoustic Emission*	55.507 kHz	51.06 kHz	92%	8%
*Cutting Force*	151.78 N	147.44 N	97.2%	2.8%
**Experimental Result 3**	**Predicted Value**	**Experimental Value**	**Accuracy**	**Uncertainty**
*Surface Roughness*	0.63 µm	0.55 µm	85.5 %	14.5%
*Motor Current*	49.93 Hz	49.96 Hz	99.9%	0.01%
*Cutting Force*	95.45 N	100.85 N	95%	5%
**Experimental Result 4**	**Predicted Value**	**Experimental Value**	**Accuracy**	**Uncertainty**
*Flank Wear*	0.132 mm	0.127 mm	96%	4%
*Temperature*	472.13 °C	486.3 °C	97%	3%
*Vibration*	50.64 Hz	50.05 Hz	99%	1%

## Nomenclature

ANOVA	Analysis of Variance
TCMS	Tool Condition Monitoring System
RSM	Response Surface Methodology
S/N	Signal to Noise Ratio
SR	Surface Roughness (µm)
*AE*	Acoustic Emission (kHz)
*F*	Cutting Force (N)
*V_B_*	Flank Wear (mm)
*Ra*	Averaged Surface Roughness (µm)
*Vb*	Vibration (Hz)
*MC*	Motor Current (Hz)
*T*	Temperature (°C)
CBN	Cubic Boron Nitride
PCD	Polycrystalline Diamond
PVD	Physical Vapor Deposition
*f*	Feed Rate (mm/rev)
*a_p_*	Depth of Cut (mm)
*v_c_*	Cutting Speed (m/min)
